# Cyanobacterial bloom causes expansion of isotopic niche areas and overlap in crustacean zooplankton

**DOI:** 10.1038/s41598-025-15061-1

**Published:** 2025-08-08

**Authors:** Wojciech Krztoń, Edward Walusiak, Elżbieta Wilk-Woźniak

**Affiliations:** Institute of Nature Conservation, Polish Academy of Sciences, al. Adama Mickiewicza 33, 31-120 Kraków, Poland

**Keywords:** Zooplankton, Copepods, Cladocerans, Cyanobacterial blooms, δ^13^C, δ^15^N, Biogeochemistry, Food webs, Limnology

## Abstract

We aimed to study how cyanobacterial blooms affect the use of the basal resources by three groups of crustacean zooplankton (calanoid and cyclopoid copepods, *Daphnia* spp.). We used measurements of naturally occurring stable isotopes of carbon (δ^13^C) and nitrogen (δ^15^N) to quantify the areas of isotopic niches (sample size-corrected standard ellipse areas; SEA_c_) of planktonic crustaceans during the pre-bloom and cyanobacterial bloom phases. In the pre-bloom phase, SEA_c_s accounted for 15.0‰^2^ in calanoid copepods, 21.2‰^2^ in cyclopoid copepods and 14.4‰^2^ in *Daphnia* spp. During the cyanobacterial bloom phase, the SEA_c_s of studied animals increased to 37.8, 27.0 and 43.6‰^2^ respectively. In addition, the overlap among the niches of the crustacean groups increased during the bloom phase compared to the pre-bloom phase. The results suggest that, despite reduced diversity of basal resources during the cyanobacterial bloom, crustaceans exhibited dietary adaptability. This involved a shift toward alternative food sources.

## Introduction

Freshwater ecosystems cover less than 1% of the Earth’s surface but constitute habitat for almost 6% of the world’s species^1^, including invertebrates, fish and plant species as well as amphibians and birds that use freshwater as important breeding habitats^2^. Furthermore, freshwaters provide various critical ecosystem services such as the supply of drinking and irrigation water, food security, recreational areas, climate-regulating carbon sequestration and much more^3,4^. Pelagic food webs of these ecosystems are supported by a vast diversity of primary producers (bacteria and algae) fixing allochthonous and autochthonous carbon, which then serve as food sources for planktonic animals^5,6^. Among the latter, planktonic crustaceans are a primary group in the process of energy flow through freshwater food webs. Thanks to diverse foraging strategies these animals are capable of grazing a variety of available sources (algae, bacteria, other animals)^7–9^ and large body sizes determine them as an attractive food for fish^10^.

Freshwater ecosystems are characterized by seasonal succession of plankton communities, which is described by the Plankton Ecology Group (PEG) model, but addresses primarily deep stratified lakes^11,12^. Functioning of shallow waterbodies in turn, is determined by their state of stability (macrophyte or phytoplankton dominance) and involves a more intense interaction between water and bottom sediment^13–15^. An integral part of this seasonal cycle is the cyanobacterial bloom phase, characteristic for late summer/ early fall, when water temperature reaches highest levels^16^. The potential toxicity and large sizes of the filaments and colonies, followed by a low nutritional value define cyanobacteria as a poor quality food for zooplankton grazers^17^. The dominance of cyanobacteria during the bloom can trigger reassembling of plankton communities^18,19^. In extreme cases (such as prolonged cyanobacterial blooms), this can lead to disruption of energy flow pathways in the food web^20^.

Various field and laboratory studies carried out so far have addressed the relationship between the ecological niche of planktonic crustaceans and the functioning of aquatic ecosystems (Reviewed by Sodré and Bozelli^21^). The main determinants of niche size in planktonic crustaceans are feeding traits, such as preferred size and type of food (phytoplankton, bacteria, animal prey), feeding efficiency and food uptake mode^22^. Although these traits perform well in characterizing community structure, quantification of niche sizes can be biased due to the gaps in existing knowledge on species characteristics^23^. However, an accurate estimation of the ecological niche occupied by the species is feasible using measurements of naturally occurring stable isotopes of carbon (δ^13^C) and nitrogen (δ^15^N). δ^13^C and δ^15^N are indicators of the origin of carbon in the diet and the trophic position of the organism, respectively^24^. Furthermore, δ^13^C and δ^15^N representing dimensions in a Hutchinsonian (n-dimensional) niche concept^25,26^ can be defined as isotopic niches and treated as a proxy for species’ ecological niches^27^. Isotopic niches are powerful tools for tracking shifts in the resource use by the organisms, which allows responses to environmental changes to be determined. A detailed analysis of species’ isotopic niches based on δ^13^C and δ^15^N, can be performed using Stable Isotope Bayesian Ellipses implemented in R Statistical Software (‘SIBER’ package;^28^). Shifts in areas of the ellipses, their relative position in δ-space (δ^13^C vs. δ^15^N biplot) and metrics of the distribution of observations provide robust information about the ecology of species (and communities)^26–28^. In a reasonable manner changes in isotopic niches of crustaceans can be used as indicators of changes in the planktonic food webs specifically during the mass cyanobacteria development.

A large biomass of inedible cyanobacteria (large, potentially toxic filaments and colonies), which dominate the phytoplankton community during blooms can trigger various grazing responses of crustacean zooplankton—the fundamental grazers in freshwater food webs^29^. In this work we aimed to study how the width of the ecological niches of three main groups of crustacean zooplankton (calanoid and cyclopoid copepods, *Daphnia* spp.) changes during the cyanobacterial bloom. We hypothesized that the areas and relative positions of isotopic niches of planktonic crustaceans change during cyanobacterial blooms. Considering the fact that observed climatic changes will force proliferation of cyanobacterial blooms globally and especially in the Northern Hemisphere^30^, a better recognition of the consequences of these phenomena on freshwater ecosystems is necessary, as long-term cyanobacterial blooms could destabilize the food web. Disruptive effects on pathways of energy flow appear to be a consequential problem. Therefore, the results of this work support the understanding of shifts in energy fluxes in freshwater food webs exposed to cyanobacterial dominance. Changes in the width of the isotopic niches of different crustacean groups can be good indicators of changes in the trophic network, allowing its stability to be assessed.

## Results

The ecosystems selected for the study were shallow waterbodies with a mean depth of 1.48 to 5.5 m and a mean water transparency ranging between 0.39 and 1.26 m, seasonally decreasing below 0.2 m. During the studied period, the mean water temperature in the waterbodies was between 15.02 and 19.48 °C (Table 1). The mean NO_3_^-^ concentration was between 1.57 and 9.29 mg/L, the mean NH_4_^+^ concentration was between 0.14 and 0.57 mg/L and the mean PO_4_^3-^ concentration was between 0.03 and 0.12 mg/L.

**Table 1:** Basic information about studied waterbodies.

Lake	Coordinates	Area [ha]	Mean depth [m]		Mean Secchi depth [m]		Mean water temperature [°C]	
			Mean	Range	Mean	Range	Mean	Range
Wołowice 1	49°59′17.6"N 19°45′01.5"E	4.19	5.2	4.5–5.9	0.68	0.15–1.1	15.02	8.3–20.2
Wołowice 2	49°59′05.5"N 19°44′35.8"E	5.33	5.5	5.3–6.0	0.67	0.5–0.9	15.98	8.6–22.3
Tyniec 1	50°01′47″ N, 19°49′39.8″ E	5.75	2.35	1.5–2.9	0.77	0.3–1.3	18.16	11–25.5
Tyniec 2	50°01′28.1″ N, 19°48′47.7″ E	8.56	1.48	1.2–1.8	0.39	0.3–0.7	18.3	10.5–25.5
Podkamycze 1	50°05′11″ N, 19°50′01.6″ E	16.82	2.2	1.8–2.5	1.26	0.4–2.4	17.56	10.2–24.7
Podkamycze 2	50°04′59.6″ N, 19°50′05.4″ E	17.28	1.86	1.7–2.3	1.02	0.2–2.3	19.48	11–25.6

In both sampling years in the studied waterbodies cyanobacterial blooms were present from mid-July to the end of October and were formed by *Aphanizomenon flos-aquae* Ralfs ex Bornet & Flahault 1886*, A. gracile* Lemmermann 1907*, Dolichospermum mendotae* (W.Trelease) Wacklin, L.Hoffmann & Komárek 2009*, D. circinale* Rabenhorst ex Bornet & Flahault) Wacklin, Hoffmann & Komárek 2009*, Microcystis aeruginosa* (Kützing) Kützing 1846 *and M. wesenbergii* (Komárek) Komárek ex Komárek 2006.

The ranges of the δ^13^C and δ^15^N values of studied animals in pre-bloom and bloom phase are presented in (Table 2). In the pre-bloom phase, the mean δ^13^C values were -33.6‰ in calanoid copepods, -31.9‰ in cyclopoid copepods and -30.7‰ in *Daphnia* spp. During the bloom δ^13^C values found in crustacean tissues were: -31.4‰ in calanoid copepods, -30.7‰ in cyclopoid copepods and -31.6‰ in *Daphnia* spp. Calanoid copepods showed the highest mean values of δ^15^N among studied animals, which were 14.2‰ and 13.7‰, in pre-bloom and bloom phase, respectively. The lowest values of δ^15^N were found in *Daphnia* spp. tissues: 11.7‰ in pre-bloom and 10.6‰ in bloom phase. The mean δ^15^N values found in the tissues of cyclopoid copepods were 13.7‰ and 12.7‰ in pre-bloom and bloom phases, respectively.

**Table 2:** Mean values and ranges of δ^13^C and δ^15^N (in ‰) measured in planktonic crustaceans in pre-bloom and bloom phase.

	δ^13^C				δ^15^N			
	Pre-bloom		Bloom		Pre-bloom		Bloom	
	Mean	Range	Mean	Range	Mean	Range	Mean	Range
Calanoida	−33.6	−38.6–−27.2	−31.4	−42.7–−23.7	14.2	11–18.7	13.7	7.22–17.8
Cyclopoida	−31.9	−36.9–−25.7	−30.7	−40.4–−22.2	13.7	9.6–19.8	12.7	7.8–15.9
*Daphnia spp.*	−30.7	−35.4–−25.1	−31.6	−42.5–−25.3	11.7	7.9–16.1	10.6	2.6–17.9

**Fig. 1:**
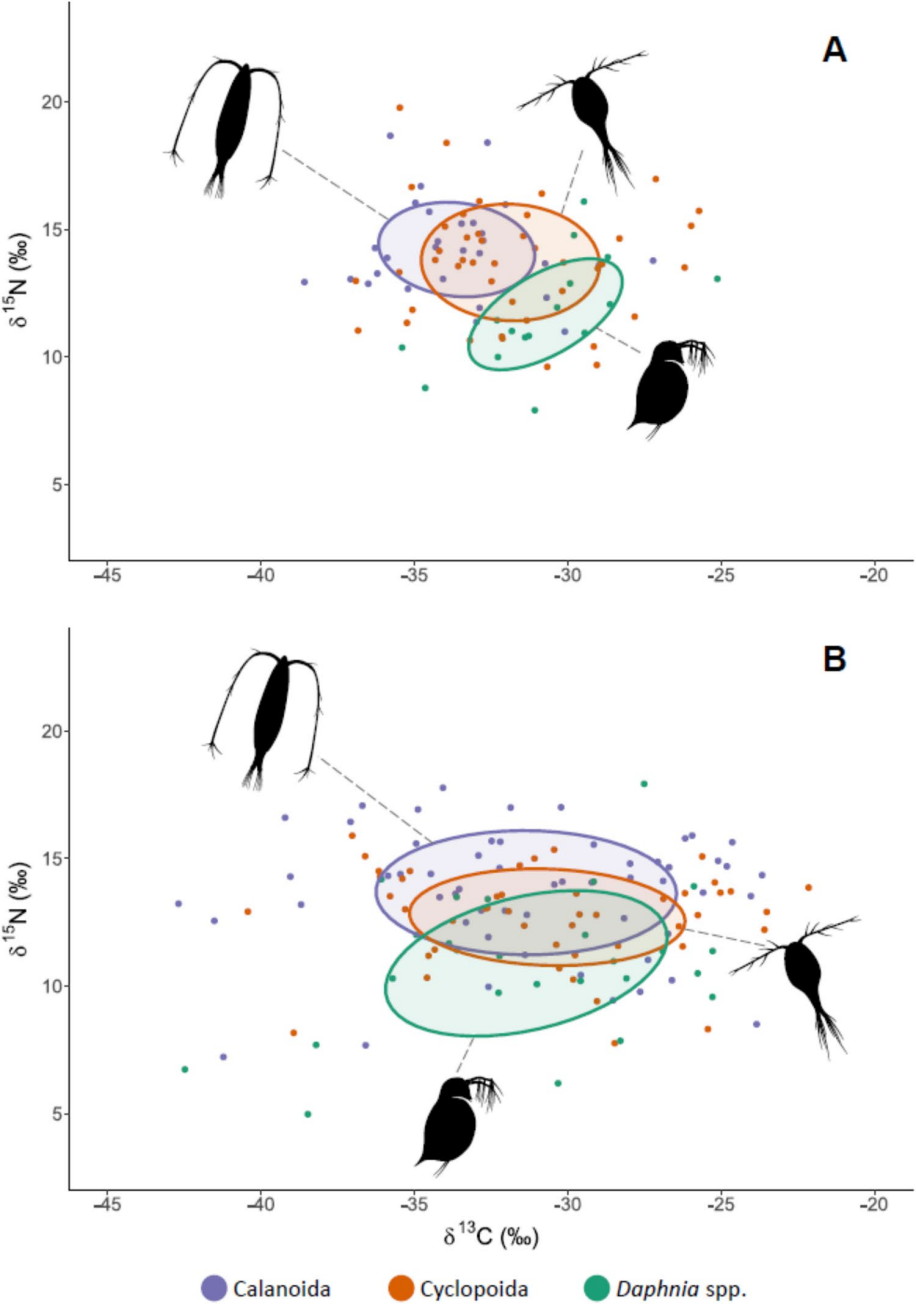
Bayesian standard ellipses corrected for small samples sizes (SEA_c_) representing core 40% isotopic niches of planktonic crustaceans during; (**A**) pre-bloom phase; (**B**) cyanobacterial bloom phase.

In the pre-bloom phase, the niches of calanoid copepods and *Daphnia* spp. were separated, as the overlap of their ellipses accounted for < 0.1% (overlapped area: 0.01 ‰^2^; Fig. 1A). The niches of cyclopoid copepods and *Daphnia* spp. showed larger overlap, accounting for 15.6% (overlapping area: 5.6‰^2^; Fig. 1A), while the overlap between the niches of calanoid and cyclopoid copepods was the highest, and reached 28.2% (overlapping area: 10.2‰^2^; Fig. 1A). During the bloom phase, the niche overlap of all planktonic crustaceans increased in all cases. The overlap of calanoid copepods and *Daphnia* spp. ellipses increased to 17% (overlaping area: 13.9‰^2^; Fig. 1B), the overlap of the niches of cyclopoid copepods and *Daphnia* spp. increased to 24.6% (overlapping area: 17.4‰^2^; Fig. 1B) and the overlap between the niches of calanoid and cyclopoid copepods increased to 35% (overlapping area: 22.7‰^2^; Fig. 1B).

We found an asymmetrical overlap between pre-bloom and bloom community SEA_c_s for all studied crustacean groups. In calanoid copepods, 91% of the pre-bloom SEA_c_ overlapped with the bloom SEA_c_, while the bloom SEA_c_ overlapped only 36% of the pre-bloom area. In cyclopoid copepods, the pre-bloom SEA_c_ overlapped 69.2% of the bloom SEA_c_, and the bloom SEA_c_ overlapped 54.2% of the pre-bloom area. In *Daphnia* spp., the pre-bloom SEA_c_ overlapped 99.7% of the bloom SEA_c_, whereas the bloom SEA_c_ overlapped only 32.9% of the pre-bloom area.

**Fig. 2:**
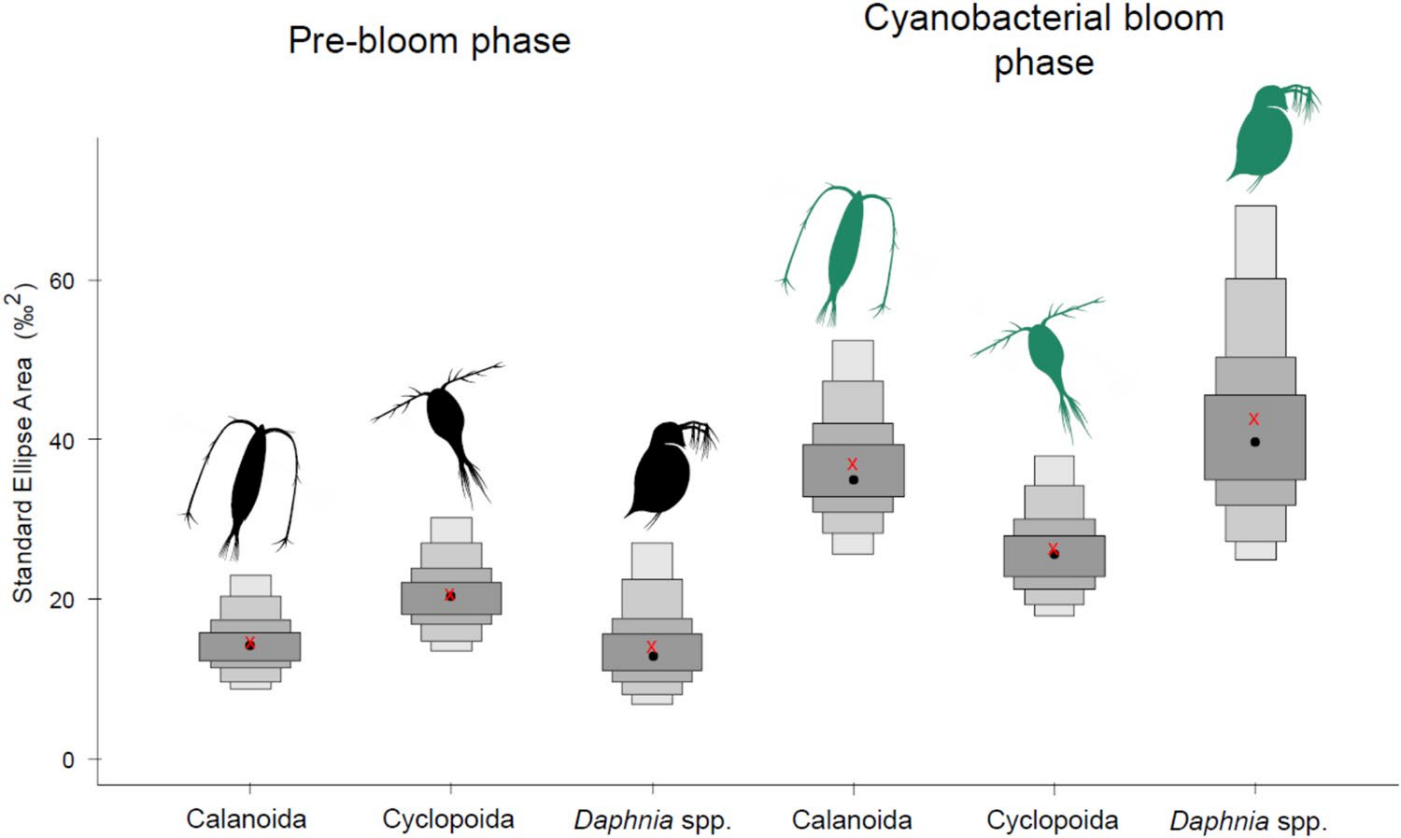
Density plots for credibility intervals (99, 95, 75, 50%) of the standard ellipse areas (SEA) for planktonic crustaceans based on stable isotope analysis. Red X represents estimated SEA_c_.

In order to analyze intra- and among- groups effect of cyanobacterial bloom on the feeding ecology of studied animals we compared the SEA_c_ calculated for each group in the pre-bloom and bloom phases. The SEA_c_s of the studied animals in both, pre-bloom and bloom phases are presented in (Fig. 2). All SEA_c_s were within the 99% credibility interval of the Bayesian estimated posterior distributions (Fig. 2 and Table 3). In the pre-bloom phase isotopic niches (SEA_c_s) were 15.0‰^2^ in calanoid copepods, 21.2‰^2^ in cyclopoid copepods and 14.4‰^2^ in *Daphnia* spp. (Fig. 2). During the cyanobacterial bloom SEA_c_ of all groups of studied animals increased, reaching 37.8‰^2^ in calanoid copepods, 27.0‰^2^ in cyclopoid copepods and 43.6‰^2^ in *Daphnia* spp. (Fig. 2).

**Table 3:** Standard Ellipse Areas (SEA_c_; in ‰^2^) of studied planktonic crustaceans in pre-bloom and bloom phase.

	Pre-bloom	Bloom
Calanoida	15.0	37.8
Cyclopoida	21.2	27.0
*Daphnia* spp.	14.4	43.6

Using Bayesian Layman metrics^28^, we found that the cyanobacterial blooms not only changed the size and relative position of the niches of studied animals but also the structure of the community (Table 4). We found that NR increased from 2.54‰ in the pre-bloom phase to 3.08‰ during the bloom phase. CR decreased from 2.9‰ in the pre-bloom phase to 0.9‰ during the bloom phase. The mean distance to centroid (CD) decreased from 1.48‰ in pre-bloom phase to 1.26‰ during the bloom phase. The mean nearest neighbor distance (MNND) decreased from 1.99‰ in pre-bloom phase to 1.55‰ during the bloom. Finally, the standard deviation of the nearest neighbor distance (SDNND) increased from 0.29‰ in the pre-bloom phase to 0.65‰ during the bloom phase.

**Table 4:** Bayesian Layman metrics calculated for planktonic crustacean communities in pre-bloom and bloom phase.

	Pre-bloom	Bloom
NR	2.54	3.08
CR	2.90	0.90
CD	1.48	1.26
MNND	2.00	1.56
SDNND	0.29	0.65

## Discussion

The ecological niches of planktonic crustaceans reflect their diverse feeding strategies, which are determined by taxon-specific functional traits. Additionally, the size of niches undergo alterations driven by changes in resource availability and/or quality^21^. By analyzing the stable isotopes of δ^13^C and δ^15^N in the tissues of three groups of planktonic crustaceans, we were able to quantify their isotopic niches and track the changes in the size and the overlap of the niches caused by cyanobacterial blooms. These shifts indicate the crustaceans’ dietary adaptability to changes in basal resource conditions during the bloom. One plausible explanation could involve ‘learning’ responses to bloom phenomena, possibly facilitated by prior exposure to recurrent cyanobacterial blooms in the studied water bodies. However, within this study, prior exposure effects were not considered.

Our results demonstrated that the general arrangement of the trophic positions of the studied crustacean groups in the food web remained unaffected by the cyanobacterial bloom. In both the pre-bloom and bloom phases, the measured δ^15^N values were highest in calanoid copepods, while the δ^15^N values of *Daphnia* spp. were the lowest among the studied groups. In both phases, the δ^15^N values of the cyclopoid copepods were intermediate between the calanoid copepods and *Daphnia* spp. Cladocerans of the genus *Daphnia* are known to rely almost exclusively on primary producers (algae and bacteria) as a food source^31^, therefore the lowest δ^15^N values indicating their lowest trophic position were to be expected as δ^15^N tends to increase with trophic level^26^. Copepods, in general, can forage on both algal and animal prey and can adjust their feeding behavior to specific environmental conditions^32–34^. For instance, the calanoid copepod *Eudiaptomus gracilis*, which is commonly found in freshwaters and is classically considered a herbivore, can effectively graze on animal prey such as protozooplankton^35,36^. Likewise, cyclopoid copepods are able to forage both on algae and animal prey^32,37^. Higher δ^15^N values in both copepod groups indicate a higher proportion of animal prey in their diet compared to *Daphnia* spp. Nevertheless, the statement that both copepod groups occupy a higher trophic level than *Daphnia* spp. is unreasonable, as the relative enrichment in δ^15^N between the groups is in most cases lower than 3‰ in most cases. According to the established trophic enrichment factors (TEFs)^38^, a relative enrichment of 3—4‰ in δ^15^N indicates placement of the studied organisms at different trophic levels. The only case when the intra-group enrichment in δ^15^N could indicate difference in trophic levels is the bloom phase, when the mean δ^15^N values of calanoid copepods were 3.1‰ higher than *Daphnia* spp. However, δ^15^N values of both groups decreased compared to the pre-bloom phase, so such inference would be an exaggeration. The limitation of this study is the lack of δ^15^N isotopic baseline for primary producers and therefore estimation of the absolute trophic level is unfeasible. Still, even if the results discussed here can be only regarded relatively, the differences in δ^15^N values among the studied groups of animals are in line with the survey of Sommer and Sommer^39^, who summarized δ^15^N differences ranging from 1–5.8‰ among comparable groups of zooplankton.

Feeding plasticity is a crucial feature that allows crustacean zooplankton adaptation to changing environmental conditions. This includes changes in availability and/or quality of basal resources as well as alteration of food particle sizes in the food web^29^, that would, for instance occur during cyanobacterial blooms^40^. We found that, compared to the pre-bloom phase, the areas of isotopic niches (SEA_c_; reflecting diet composition) of calanoid copepods, cyclopoid copepods and *Daphnia* spp. increased during the bloom phase indicating diversification of food sources. This conclusion is supported by the fact that the niches were extended mainly along the δ^13^C dimension in the isotopic biplot, which can be the consequence of fractionation of δ^13^C values among different groups of algae and other components of the food web^41^. Our results corroborate findings of Ger et al*.*^34^, who demonstrated that the calanoid copepod species *Notodiaptomus iheringi* incorporated cyanobacteria into its diet as eukaryotic prey availability declined, indicating dietary diversification under resource limitation. In contrast, the same experiment^34^ showed that cyclopoid copepods exhibited lower grazing rates when exposed to cyanobacterial biomass, however their role in filament shortening was highlighted. Even though cyanobacteria are not a preferred food source, they can still constitute a considerable proportion of food ingested by cyclopoid copepods (up to c.a. 40%) as demonstrated by Tõnno et al.^42^, who analyzed phytoplankton pigments in gut contents of planktonic crustaceans. In addition to the ability to feed on various forms of phytoplankton, copepods can forage on animal prey^35,43^, which is likely an another reason for the increasing copepod niche areas during the bloom. According to the results presented by Kosiba and Krztoń^8^, predation on large, free-swimming protozoans tended to increase in cyclopoid copepods during the cyanobacterial bloom. Similarly, calanoid copepods were shown to switch from cyanobacteria to ciliate prey in a mesocosm experiment^44^. Cladocerans of the genus *Daphnia* are filter-feeders with limited particle selection ability^45,46^, and a wide spectrum of potential food sources, including heterotrophic bacteria, phytoplankton and occasionally animal prey^7,47–52^. During the bloom, toxic and/or hard-to-handle cyanobacteria particles can negatively affect *Daphnia* and lead to a decline in their populations^53,54^. However, the cyanobacterial bloom can promote the development of organisms such as heterotrophic bacteria, ciliates or chytrid parasites, constituting ‘microbial loop’^55^. These microbial loop components are demonstrated to enhance *Daphnia* resilience to cyanobacterial blooms^56,57^ and can explain the increase of SEA_c_ of *Daphnia* which was found in our samples during the cyanobacterial blooms.

Planktonic crustaceans use distinct, taxon-specific mechanisms of food uptake effective against diverse food particles and therefore the impact of coexisting groups on the food web is comprehensive. For instance, in the case of phytoplankton, larger particles are suppressed by copepods and smaller ones by cladocerans^9,39^. This implies a robust niche partitioning among studied groups of animals. Our results are partially consistent with this statement, specifically in the case of calanoid copepods and *Daphnia* spp. in the pre-bloom phase, when the isotopic niches of the animals were not overlapping. Also in this phase, a notable portion of the cyclopoid SEAc overlapped with both calanoid copepods and *Daphnia* spp. This suggests that cyclopoid copepods exhibit high opportunism in feeding compared to calanoid copepods and *Daphnia* spp.^37^. Finally, our results corroborate with the study of Santer et al*.*^58^ who demonstrated stable isotope-based evidence of cyclopoid copepods foraging on a wide spectrum of available food sources.

During the phase of cyanobacterial bloom we observed an increased overlap in the isotopic niches of studied animals, suggesting that planktonic crustaceans shared available basal resources more evenly. The increased abundance of cyanobacteria (considered inedible or at least low-quality food) followed by a decreasing availability of eukaryotic phytoplankton, could have induced physiological and behavioral responses in crustacean grazers (enhanced tolerance and selective feeding;^17^) causing homogenization of their ecological niches. This conclusion is supported by Josué et al*.*^59^, who found that cyanobacterial dominance altered the distribution of functional traits and reduced the functional dispersion of the zooplankton community, leading to an increase in the overlap of zooplankton niches. Mentioned study employed a trait-based approach that infers ecological responses from a priori defined functional traits of species, providing complementary but static view of niche dynamics^60^. Our study supports the conclusions of Josué et al*.*^59^, with the evidence from measurements of stable isotopes (δ^13^C, δ^15^N) which are robust tracers of animals’ isotopic (ecological) niche spaces^61^.

Beyond group-specific realized niche characteristics, it is also important to consider community-level metrics to capture broader ecological patterns. We found that the NR of the community increased slightly during the bloom compared to the pre-bloom phase, indicating that the trophic length of the community marginally increased^62^. This suggests a minor diversification of feeding strategies involving more trophic levels of studied animals^62^ and is consistent with the fact that isotopic niches (i.e. SEA_c_s) of all studied groups of animals increased during the cyanobacterial bloom. Another explanation is the seasonal decrease in the δ^15^N baseline caused by the production of organic matter (cyanobacterial biomass) that is depleted in heavy nitrogen isotope (^15^N) at the start of the bloom. The biomass is then utilized by planktonic crustaceans (directly or indirectly) and δ^15^N in their tissues decreases^63^. This explanation supports our findings, as we observed decreased mean δ^15^N values in the studied animals during the bloom phase.

Thanks to various adaptations cyanobacteria can outcompete eukaryotic phytoplankton and develop extensive amounts of biomass. This can lead to disruption of the energy transfer pathways in the food webs^20^. Bayesian Layman metrics^28,62^ calculated for crustacean communities confirm this conclusion. We have observed a considerable decrease in CR (by 2‰) during the bloom phase, which implies a decline of diversity of basal resources^28^. Furthermore, CD and MNND also decreased, suggesting that the overall trophic diversity (i.e. utilized resources) of studied animals also decreased. Finally, SDNND increased during the bloom phase, suggesting that the evenness of species distribution in the δ^13^C vs. δ^15^N biplot was reduced and therefore indicating higher trophic proximity of the studied animals^28,62^. Cyanobacterial blooms disrupt important ecosystem processes such as energy flow through the food web. However, some aspects, such as the fate of cyanobacterial carbon and alternative pathways of energy flow, are still understudied^64^. Our results show that in response to the cyanobacterial blooms, resources used by the planktonic crustacean community (grazers) became limited, leading to diminishing their role as conduits of energy flow.

## Conclusions

Altogether, the quantification of ecological (isotopic) niches based on δ^13^C and δ^15^N measurements allowed for the conclusion that cyanobacterial blooms have complex effects on crustacean zooplankton, which are likely to amplify to the ecosystem level. Although the areas of the niches of particular planktonic crustaceans groups increased, the overall capacity of the whole community decreased. Obtained results indicate a collapse in the diversity of basal food resources and a reduction in the trophic differentiation within the zooplankton community, which likely impairs energy transfer efficiency in freshwater food webs.

To our knowledge, this is the first study to demonstrate that cyanobacterial blooms can simultaneously expand isotopic niche areas and increase niche overlap among coexisting crustacean zooplankton in shallow freshwater ecosystems. The use of stable isotope measurements is advantageous in ecological studies of planktonic animals and brings capacity of robust inference on the ecological processes happening in freshwater ecosystems.

## Methods

The study was carried out in six shallow, eutrophic waterbodies located in southern Poland, in the area of Kraków: four oxbow lakes (Wołowice 1, Wołowice 2, Tyniec 1, Tyniec 2) and two artificial ponds (Podkamycze 1, Podkamycze 2). The waterbodies were selected for this study on the basis of previous regular monitoring studies carried out since 2014 (Tyniec 1, Tyniec 2, Podkamycze 1, Podkamycze 2) and several field surveys (Wołowice 1, Wołowice 2). These examinations allowed identification of the waterbodies where cyanobacterial blooms occur annually, ensuring that the exposure and adaptation histories of planktonic crustacean communities are comparable. Basic information on studied waterbodies are presented in (Table 1). Samplings were conducted bi-weekly in the period from April to October in 2019 and 2020. The depth of each waterbody and water transparency were measured in situ with use of Secchi disc. The samples were collected from the central point of each waterbody with the use of a 5L sampler, and covered the whole water column (sampling interval: 0.5 m starting from the surface to the bottom of the waterbody). The physio-chemical parameters of water included temperature, pH, conductivity and ion concentration. Water temperature, pH and conductivity were measured using YSI ProDSS sonde (YSI Incorporated, Xylem Analytics; Yellow Springs, OH, USA). Ion concentrations were determined in the laboratory of the Institute of Nature Conservation, Polish Academy of Sciences using Dionex ion chromatograph (DIONEX, IC25 Ion Chromatograph; ICS-1000, Sunnyvale, CA, USA).

The samples for the phytoplankton composition were concentrated over a plankton net with mesh size of 10 μm from an initial volume of 10L, and fixed with Lugol’s iodine solution. Qualitative analyzes of the phytoplankton were conducted using a Zeiss Jenaval (Oberkochen, Germany) light microscope (magnifications between 40 × and 400 ×). Phytoplankton identification was carried out based on taxonomic keys listed in^65^ and supplemented by Komarek^66^.

Samples for the measurements of stable isotopes of carbon (δ^13^C) and nitrogen (δ^15^N) in crustaceans were collected in the field using vertical hauls of a 50 µm planktonic net, and then sorted in the laboratory using a light microscope. The material was divided into pre-bloom and cyanobacterial bloom phase, based on field observations and confirmed with microscopic analyses. The following definition of ‘cyanobacterial bloom’ was applied: “a marked visible discoloration of the water that is caused (predominantly) by cyanobacteria”^20^. In the pre-bloom phase 29 samples of calanoid copepods, 43 samples of cyclopoid copepods and 16 samples of *Daphnia* spp. were isolated. During the bloom phase 56 samples of calanoid copepods, 47 samples of cyclopoid copepods and 27 samples of *Daphnia* spp. were collected. Single samples consisted of at least 100 adult specimens. A total of 218 samples were analyzed including 88 samples in the pre-bloom phase and 130 samples in the bloom phase. The analyses of stable isotopes were performed at the Stable Isotope Laboratory of the Institute of Geological Sciences, Polish Academy of Sciences, Warsaw, Poland, using a Flash 1112 HT elemental analyzer coupled to Delta V Advantage IRMS (Thermo Scientific; Waltham, Massachusetts, USA) with continuous flow mode and Helium (He) carrier gas. Stable isotope values are reported in delta (δ) notation as parts per thousand (‰) deviation from Viena PeeDee Blemnite (VPDB) for δ^13^C and atmospheric air for δ^15^N. The results of duplicate analysis were calibrated with three international standards USGS 41a (L-glutamic acid, δ^13^C =  + 36.55 ± 0.08 ‰, δ^15^N =  + 47.55 ± 0.15 ‰), USGS 40 (L-glutamic acid, δ^13^C = -26.389 ± 0.042 ‰, δ^15^N = -4.5 ± 0.1 ‰) and IAEA 600 (caffeine, δ^13^C = -27.771 ± 0.043 ‰, δ^15^N =  + 1.0 ± 0.2 ‰). As an internal laboratory standard to control for instrument drift, we used USGS 65 (glycine, δ^13^C = –20.29 ± 0.04 ‰, δ^15^N + 20.68 ± 0.06 ‰). Based on the laboratory standard measurements, measured in each cycle run, the precision (1σ) and reproducibility were generally better than ± 0.1‰ for δ^13^C and ± 0.3‰ for δ^15^N, respectively. To calculate the C/N ratio the same standards were used, the C/N ratio was estimated with accuracy ± 0.1. Planktonic crustaceans contain lipids, which are depleted in ^13^C leading to lower δ^13^C values. Therefore δ^13^C were corrected for differential lipid content using the elemental C:N ratio according to Syväranta & Rautio^67^.

To calculate the isotopic niche space and the overlap of the isotopic (ecological) niche spaces among studied planktonic crustaceans we employed Stable Isotope Bayesian Ellipses from ‘SIBER’ R-environment package^28^. Isotopic niches of planktonic crustaceans understood as Standard Ellipses Areas (SEA) represented core ecological niches, and were calculated as Corrected (for small sample size) Standard Ellipses Areas (SEA_c_) based on 40% of measurements of stable isotopes (δ^13^C and δ^15^N) of studied animals. Credibility Intervals for SEA_c_ were calculated as Bayesian equivalent (SEA_b_), in order to analyze the uncertainty around ellipses and evaluate their variability^28^. The area of the standard Bayesian ellipses is reported in ‰^2^ (permille squared), as both δ^13^C and δ^15^N values are expressed in permil (‰), and the ellipse area is calculated from the covariance matrix of these values. The resulting unit, ‰^2^, accurately reflects the dispersion in isotopic space and is standard in isotopic niche analysis.

The Bayesian Layman metrics: group mean δ^15^N range (NR; indicating the trophic length of the studied community), group mean δ^13^C range (CR, indicating the diversity of basal resources for the studied community), group mean nearest neighbour distance (MNND; indicator of the density and clustering of species within the studied community), intra-group standard deviation of the nearest neighbour distance (SDNND; indicator of the evenness of spatial density and packing of species in the studied community) were calculated using ‘SIBER’ package, according to Jackson et al.^28^.

## Data Availability

Data can be accessed at https://github.com/wmkrzt/iso_niches_crustacea.git.
